# The influence of human presence and footprint on animal space use in US national parks

**DOI:** 10.1098/rspb.2025.1013

**Published:** 2025-07-30

**Authors:** Kaitlyn M. Gaynor, Forest P. Hayes, Kezia Manlove, Nathan Galloway, John F. Benson, Michael J. Cherry, Clinton W. Epps, Robert J. Fletcher Jr, John Orrock, Justine A. Smith, Christina Aiello, Jerrold L. Belant, Joel Berger, Mark Biel, Jill Bright, Joseph Bump, Michael Burchett, Carson Butler, Jennifer Carlson, Eric K. Cole, Neal Darby, Erin Degutis, Sarah Dewey, Pete Figura, Thomas D. Gable, Jeff Gagnon, Danielle M. Glass, Jennifer R. Green, Kerry Gunther, Mark A. Haroldson, Kent R. Hersey, Brandon P. Holton, Austin T. Homkes, Sarah R. Hoy, Debra Hughson, Kyle Joly, Ryan Leahy, Caitlin Lee-Roney, Rob Lester, Dan MacNulty, Michael Magnuson, Daniel Martin, Rachel Mazur, Seth Moore, Elizabeth K. Orning, Katie Patrick, Rolf O. Peterson, Lynette Potvin, Paige R. Prentice, Seth P.D. Riley, Mark C. Romanski, Annette Roug, Jeff A. Sikich, Nova Simpson, William Sloan, Douglas W. Smith, Mathew Sorum, Scott Sprague, Daniel Stahler, John Stephenson, Thomas R. Stephenson, Janice Stroud-Settles, Frank T. van Manen, John A. Vucetich, Kate Wilmot, Steve Windels, Tiffany M. Wolf, Paul C. Cross

**Affiliations:** ^1^ Department of Zoology and Botany, The University of British Columbia, Vancouver, British Columbia V6T 1Z4, Canada; ^2^ Fish, Wildlife, and Conservation Biology, Colorado State University, Fort Collins, CO 80523, USA; ^3^ Wildland Resources, Utah State University, Logan, UT 84322, USA; ^4^ Biological Resources Division, National Park Service Natural Resource Stewardship and Science Directorate, Fort Collins, CO 80525, USA; ^5^ School of Natural Resources, University of Nebraska-Lincoln, Lincoln, NE 68588, USA; ^6^ Texas A&M University Kingsville Caesar Kleberg Wildlife Research Institute, Kingsville, TX 78363, USA; ^7^ Fisheries, Wildlife, and Conservation Sciences, Oregon State University, Corvallis, OR 97331, USA; ^8^ Department of Zoology, Conservation Research Institute, University of Cambridge, Cambridge CB2 3QZ, UK; ^9^ Integrative Biology, University of Wisconsin-Madison, Madison, WI 53706, USA; ^10^ Wildlife, Fish, and Conservation Biology, University of California Davis, Davis, CA 95616, USA; ^11^ Fisheries and Wildlife, Michigan State University, East Lansing, MI 48824, USA; ^12^ Global Programs, Wildlife Conservation Society, Bronx, NY 10460, USA; ^13^ Glacier National Park, National Park Service, West Glacier, MT 59936, USA; ^14^ Region IV, Arizona Game and Fish Department, Phoenix, AZ 85086, USA; ^15^ Fisheries, Wildlife, and Conservation Biology, University of Minnesota, Saint Paul, MN 55108, USA; ^16^ Mojave National Preserve, National Park Service, Barstow, CA 92311, USA; ^17^ Grand Teton National Park, National Park Service, Moose, WY 83012, USA; ^18^ California Department of Fish and Wildlife, Redding, CA 96001, USA; ^19^ US Fish and Wildlife Service, Jackson, WY 83001, USA; ^20^ National Park Service, Yosemite National Park, CA 95389, USA; ^21^ California Department of Fish and Wildlife, West Sacramento, CA 95605, USA; ^22^ Wildlife Contracts Branch, Arizona Game and Fish Department, Phoenix, AZ 85086, USA; ^23^ California Department of Fish and Wildlife, Bishop, CA 93514, USA; ^24^ Department of Geography, The University of British Columbia, Vancouver, British Columbia V6T 1Z4, Canada; ^25^ National Park Service, Yellowstone National Park, WY 82190, USA; ^26^ Northern Rocky Mountain Science Center, Interagency Grizzly Bear Study Team, U.S. Geological Survey, Bozeman, MT 59715, USA; ^27^ Utah Division of Wildlife Resources, Salt Lake City, UT 84116, USA; ^28^ Science and Resource Management, National Park Service, Grand Canyon, AZ 86023, USA; ^29^ Fisheries, Wildlife, and Conservation Biology, University of Minnesota Twin Cities, Minneapolis, MN 55108, USA; ^30^ College of Forest Resources and Environmental Science, Michigan Technological University, Houghton, MI 49931, USA; ^31^ Yukon-Charley Rivers National Preserve, National Park Service, Fairbanks, AK 99709, USA; ^32^ California Department of Fish and Wildlife, Rancho Cordova, CA 95670, USA; ^33^ Resource Management, Lassen Volcanic National Park, National Park Service, Mineral, CA 96063, USA; ^34^ National Park Service Midwest Region, Omaha, NE 68102, USA; ^35^ Organ Pipe Cactus National Monument, National Park Service, Ajo, AZ 85321, USA; ^36^ National Park Service Northeast Region, Philadelphia, PA 19107, USA; ^37^ Grand Portage Band of Lake Superior Chippewa, Grand Portage, MN 55605, USA; ^38^ College of Environmental Science and Forestry, State University of New York, Syracuse, NY 13210, USA; ^39^ Isle Royale National Park, National Park Service, Houghton, MI 49931, USA; ^40^ Santa Monica Mountains National Recreation Area, National Park Service, Thousand Oaks, CA 91362, USA; ^41^ State of Alaska Department of Fish and Game, Palmer, AK 99645, USA; ^42^ Environmental Services, Nevada Department of Transportation, Carson City, NV 89712, USA; ^43^ Death Valley National Park, National Park Service, Death Valley, CA 92328, USA; ^44^ Southeast Utah Group, National Park Service, Moab, UT 84532, USA; ^45^ Yellowstone Center for Resources, Yellowstone National Park, WY 82190, USA; ^46^ Sierra Nevada Bighorn Sheep Recovery Program, California Department of Fish and Wildlife, Bishop, CA 93514, USA; ^47^ Zion National Park, National Park Service, Springdale, UT 84767, USA; ^48^ Northern Rocky Mountain Science Center, U.S. Geological Survey, Bozeman, MT 59715, USA; ^49^ Voyageurs National Park, National Park Service, International Falls, MN 56649, USA; ^50^ Department of Veterinary Population Medicine, University of Minnesota Twin Cities, Saint Paul, MN 55108, USA

**Keywords:** animal movement, functional response, human footprint, human-wildlife interactions, protected areas, recreation, Resource Selection Function

## Abstract

Given the importance of protected areas for biodiversity, the growth of visitation to many areas has raised concerns about the effects of humans on wildlife. In 2020, the COVID-19 pandemic led to temporary closure of national parks in the United States, offering a pseudonatural experiment to tease apart the effects of permanent infrastructure and transient human presence on animals. We compiled GPS tracking data from 229 individuals of 10 mammal species in 14 parks and used third-order hierarchical resource selection functions to evaluate the influence of the human footprint on animal space use in 2019 and 2020. Averaged across all parks and species, animals avoided the human footprint, whether the park was open or closed. However, although animals in remote areas showed consistent avoidance, on average those in more developed areas switched from avoidance to selection when protected areas were closed. Findings varied across species: some responded consistently negatively to the footprint (wolves, mountain goats), some positively (mule deer, red fox) and others had a strong exposure-mediated response (elk, mountain lion). Furthermore, some species responded more strongly to the park closure (black bear, moose). This study advances our understanding of complex interactions between recreation and wildlife in protected areas.

## 1. Background

As human disturbance expands globally [1], protected areas are a critical tool for conserving many animal species that are sensitive to humans and require large swathes of intact habitat [2–4]. However, in many protected areas, wildlife conservation must be balanced with other management objectives, including human uses [5,6]. Nature-based recreation and tourism in protected areas are of meaningful cultural and economic importance [7], and non-extractive activities like hiking, camping, picnicking and sightseeing are some of the land use types most compatible with wildlife conservation [8]. Nonetheless, both transient human activity and permanent recreation infrastructure (henceforth, the ‘human footprint’) in protected areas can shape animal behaviour, with potential population-level consequences [9,10]. Indeed, meta-analyses have revealed negative effects of recreation on species occupancy [11] and richness [12].

The risk disturbance hypothesis posits that wild animals often perceive humans as risky, even in the absence of a direct threat [13,14]. Animals frequently exhibit anti-predator behaviour in response to anthropogenic stimuli [15] and adjust their patterns of movement [16] and daily activity [17] to avoid people in space and time. Within protected areas, animals frequently avoid hotspots of human activity such as trails, campgrounds, buildings and roads [18–20]. The effects of infrastructure on space use typically extend beyond its physical footprint, suggesting that avoidance is related in part to risk perception, not just poor habitat quality in developed areas [21]. These behavioural responses can reduce the amount of habitat that is effectively available to animals and can have costly physiological consequences that compromise individual survival and reproduction, with potential implications for population demography and persistence [14,22].

While many animals avoid human activity and infrastructure, the effects of human disturbance on patterns of space use vary among species and individuals [8,23]. Some animals may be attracted to anthropogenic resource subsidies [24] or find refuge from predators [25]. Within a given population, individuals that are regularly exposed to benign human disturbance may habituate to human infrastructure and other anthropogenic stimuli [26,27]. Rather than avoid disturbance, these animals may instead exhibit neutral or positive associations with disturbance, to take advantage of natural or anthropogenic resources in these areas [28,29]. Previous exposure to humans may therefore predict individual variation in the effects of disturbance on animal space use and behaviour. Habituation may facilitate human–wildlife coexistence, but it may also foster human–wildlife conflict and threaten human property and safety [30,31].

The effects of direct human presence and human footprint on animal space use may be opposing or interacting, given the relative benefits and risks associated with each [23,32]. Given their strong correlation, it can be difficult to tease apart their effects (but refer to [22,33,34]). However, in the spring of 2020, the COVID-19 pandemic disrupted global human activity, leading to widespread restrictions on movement of people and access to public spaces. Under the most unfortunate of circumstances, many ecologists leveraged this ‘Anthropause’ as a pseudonatural experiment [35,36]. Global syntheses revealed that background levels of human development mediated the effects of lockdowns on animal activity on camera traps [37] and movement trajectories [38]. Within protected areas, the pause of outdoor recreation was linked to changes in spatiotemporal patterns of wildlife activity [39–41].

Here, we present a multispecies study of mammal habitat selection in protected areas in the US National Park Service (NPS) system, which experienced an abrupt and controlled cessation of visitation during the pandemic. Since its inception, the NPS has been tasked with the dual mandate of conserving wild places and wildlife, while providing recreation opportunities for visitors [42]. Recently, the rapid growth of visitation to many parks has raised concerns about the effects of human infrastructure and activity on the distribution, behaviour and demography of animal populations [43,44]. We leveraged the pandemic protected area closures to enhance our understanding of these effects. Our goals were to quantify baseline responses of large mammals to human disturbance in NPS protected areas, quantify changes in patterns of avoidance of or selection for the human footprint during park closures, and evaluate how responses to the interacting effects of human presence and footprint varied across populations, species and individual animals.

**Figure 1.**
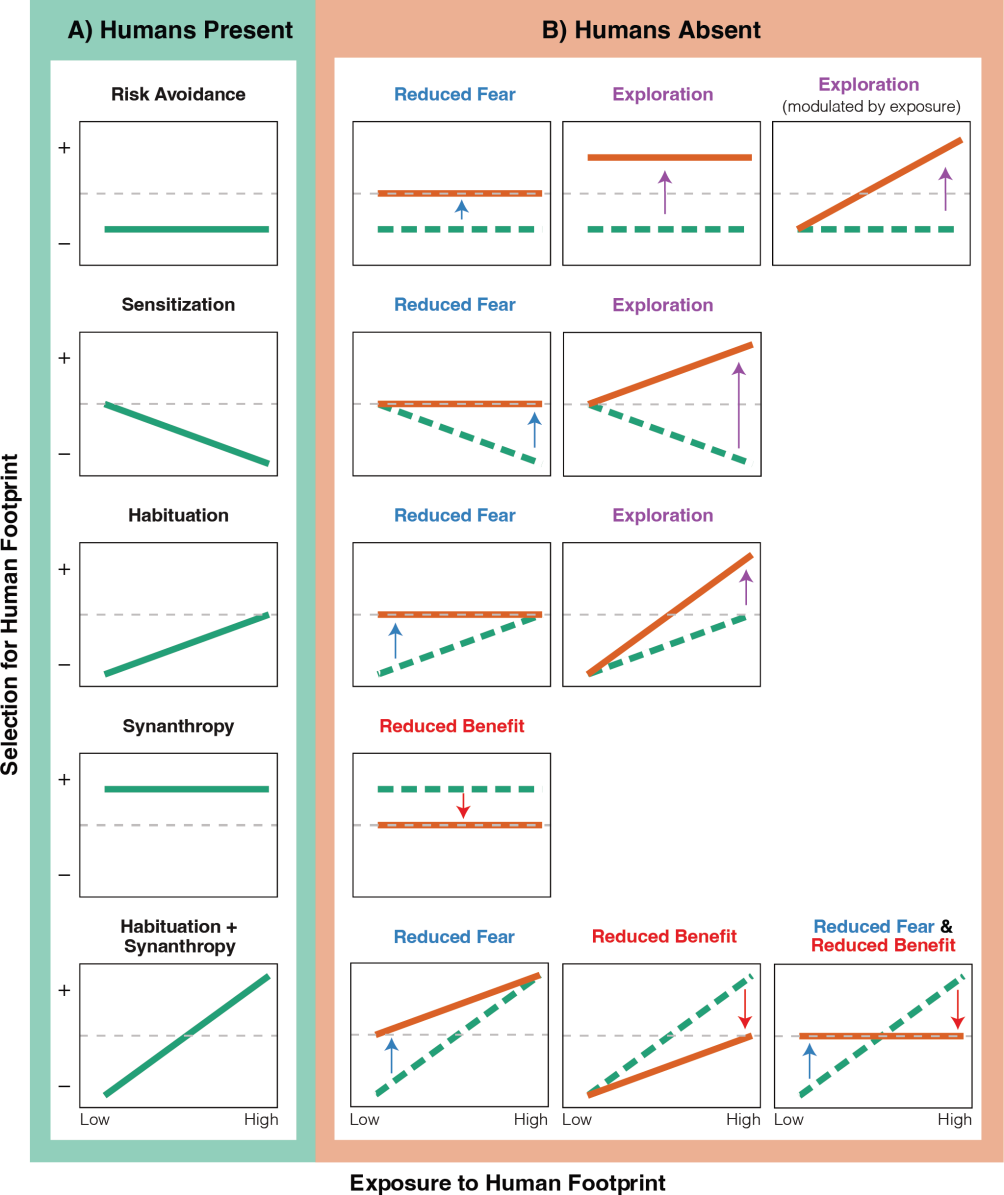
Predicted individual variation in responses of animals to human footprint and human presence (functional response hypotheses). For all plots, the *x*-axis represents an individual animal’s exposure to the human footprint (i.e. infrastructure or built environment in its home range), where a low value corresponds to an animal with little footprint in its home range (i.e. more remote areas) and a high value corresponds to an animal with high footprint in its home range (i.e. more developed areas). A negative value on the *y*-axis represents avoidance of the human footprint, and a positive value represents preferential use of areas with greater human footprint. (A) Predicted baseline responses of animals to the human footprint, during periods of typical human presence. Some animal populations may consistently avoid the footprint (Risk Avoidance), and others may select for it (Synanthropy). Animals with home ranges in more developed areas may avoid it less (Habituation) or more (Sensitization) than those in more remote areas. A combination of habituation and synanthropy may result in avoidance by animals in remote areas, and selection by animals in developed areas (Habituation+Synanthropy). (B) A subset of predicted effects of human absence (i.e. during protected area closure) on animal selection for human footprint. The removal of humans from the human footprint may reduce fear for animals that typically avoid it (Reduced Fear; blue text), or may reduce benefit for animals that prefer it (Reduced Benefit; red text), resulting in more neutral selection for the footprint. Animals may also explore areas with high human footprint in the absence of humans to take advantage of otherwise unutilized habitat, leading to a shift from negative or neutral selection for the footprint to positive selection (Exploration; purple text). Changes in selection may vary depending on the level of development in an animal’s home range.

We tested the overarching hypothesis that the human footprint and human presence interact to shape patterns of habitat selection by wild animals, as animals balance perceived risk from human activity with potential benefits offered by the human footprint. Under conditions of typical visitation, we predicted that animals would generally avoid the footprint (figure 1A: Risk Avoidance). Such responses should be most common in species sensitive to disturbance. Alternatively, we predicted that animal populations that gained some benefit from humans (e.g. food subsidies, refuge from predation) would preferentially use developed areas (figure 1A: Synanthropy). Furthermore, given that exposure to humans may influence an individual animal’s perceived risk of humans, we hypothesized that exposure would affect selection for, or against, the human footprint—in other words, a functional response between selection and availability. This functional response could take varying forms: animals that were more exposed to the human footprint might avoid the footprint less than those with home ranges in areas with a lower human footprint owing to habituation (figure 1A: Habituation) or might instead avoid it more owing to sensitization (figure 1A: Sensitization). Sensitization should be more common in populations that experience harassment and deterrents, while habituation should be more common in populations that do not experience conflict and that are synanthropic.

We predicted that these baseline responses to the human footprint in 2019 (figure 1A) would then be altered in 2020 when protected areas were closed to people (figure 1B). We predicted that responses to the human footprint would weaken, as human presence no longer posed a perceived threat (figure 1B: Reduced Fear) or provided benefit (figure 1B: Reduced Benefit). We also predicted that animals may select areas with higher human footprint in the absence of people, to take advantage of resources that otherwise went unexploited, including trails that would otherwise be used by humans (figure 1B: Exploration). Finally, we predicted that individual exposure to the human footprint would mediate the magnitude of response to protected area closures. Animals that were most exposed to human disturbance may display the strongest changes in space use in response to closures, as they would experience the largest diminution in human presence and therefore largest change in perceived risk or benefit associated with humans. Alternatively, if these animals were more habituated to human presence, they would instead display more muted responses to park closures.

## 2. Methods

We quantified patterns of mammal habitat selection in relation to the human footprint in US NPS protected areas during the park closures in 2020 and an equivalent time period with typical visitation in 2019. We used resource selection functions (RSFs), which compare locations used by an animal with those available in its home range, including an aggregate measure of human footprint as our primary covariate of interest. We then evaluated how patterns of selection or avoidance of the human footprint varied with baseline exposure to humans, and across species and protected areas, and how these relationships changed when these areas were closed to humans. We conducted all analyses in R v. 4.4.0 [45].

### (a) Data preparation

**Figure 2.**
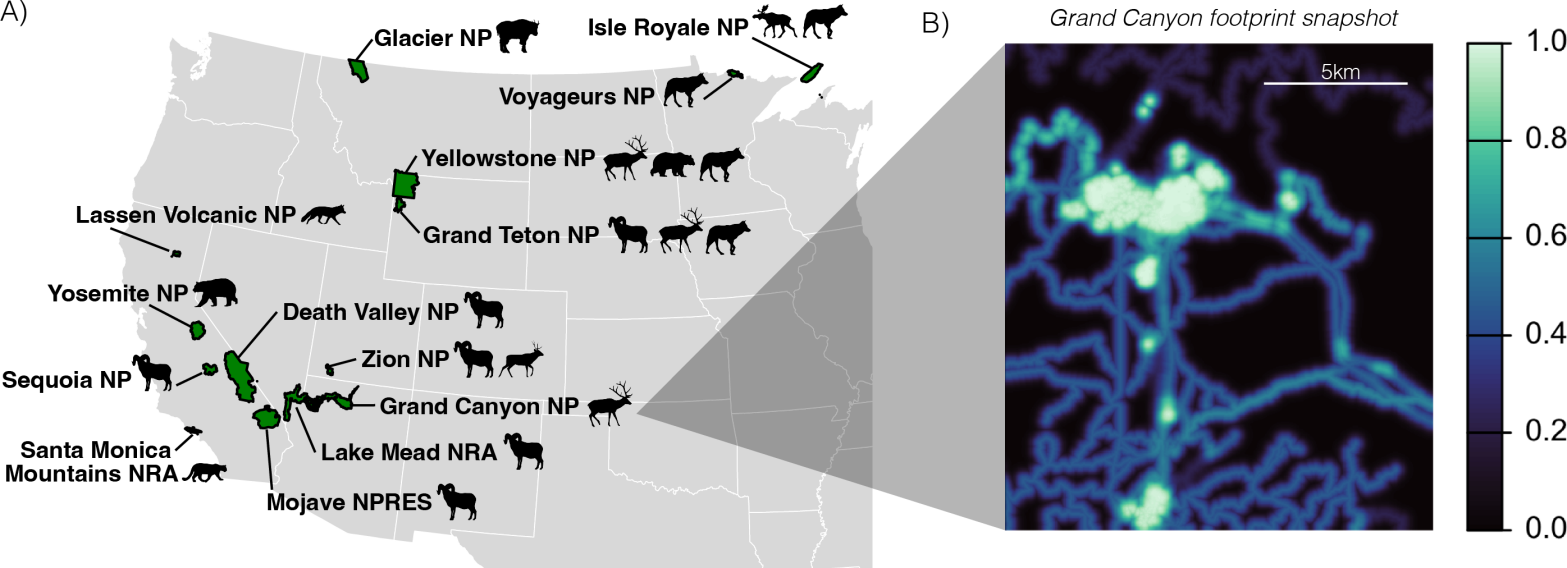
(A) Locations of all National Park Service sites (NP = park, NRA = National Recreation Area, NPRES = National Preserve) included in our study of animal movement in response to the human footprint and park closure. Animal vector silhouettes represent the study species in each park. (B) An example of the human footprint index that we generated to quantify the built environment in the study areas, mapped for the Grand Canyon National Park study area, where a value of 1.0 indicates the highest levels of human footprint. Base map in (A) was created with R package ‘maps’ [46].

We compiled GPS collar data from 2019 and 2020 for large mammals in National Parks (NP), Recreation Areas (NRA), Preserves (NPRES) and Monuments (NM) with an area >100 km^2^, managed by the US NPS (henceforth, ‘parks’). These data represented 14 parks and 20 populations of 10 species (figure 2; electronic supplementary material, table S1). The species included five carnivores (grey wolf, *Canis lupus*; mountain lion, *Puma concolor*; black bear, *Ursus americanus*; grizzly bear, *Ursus arctos;* and red fox, *Vulpes vulpes*) and five ungulates (moose, *Alces alces*; elk, *Cervus canadensis*; mule deer, *Odocoileus hemionus*; mountain goat, *Oreamnos americanus*; and bighorn sheep, *Ovis canadensis*). For most species, we only had data from a single park, with the exception of bighorn sheep (six parks), wolf (four parks) and elk (three parks). Across parks, there was a total of 229 unique individual animals tracked in one or both years, with 117 individuals tracked in both years. Given low sample sizes, we included all individuals in our population-level analyses, and we conducted a sensitivity analysis to confirm that overall findings remained consistent when only including data from individual animals collared in both years (refer to electronic supplementary material).

Prior to analysis, we removed locations that likely represented GPS error, as described in electronic supplementary material, methods. Fix intervals of the raw GPS data ranged from 30 min to 24 h. For data at higher sampling rates, we resampled locations to a 4 h fix interval to reduce spatiotemporal autocorrelation and pseudoreplication, while maintaining original fix intervals for data with lower sampling rates (electronic supplementary material, table S2). We also conducted a sensitivity analysis with a fix interval of 24 h to further ensure that our findings were robust (refer to electronic supplementary material). We clipped all GPS data to the time periods of interest (2020 park closure and 2019 equivalent dates). We identified park closure dates from the NPS website and confirmed these dates with data contributors (electronic supplementary material, table S1). We were conservative, choosing the dates that the most restrictive closures were put in place and lifted. The mean closure duration was 58 days, ranging from 31 to 83 days. We removed any individual-years with insufficient data, with more than half of locations outside the protected area, or with limited human footprint in the animal’s home range, and we filtered out points from migration and hibernation events (refer to electronic supplementary material).

### (b) Spatial covariates

To facilitate inference across parks and systems, we created an aggregate measure of the human footprint (figure 2B), akin to other widely used but coarser metrics like the Human Footprint Index [1] and the Global Human Modification Index [47]. We followed a similar approach to develop an index that best captured the finer spatial scale of infrastructure in protected areas and included five features of interest: roads, trails, car parks, buildings and campgrounds. We downloaded vector shapefiles for each feature from a combination of the publicly available NPS Official Service-wide Datasets [48] and OpenStreetMap [49] and created an aggregate measure of the human footprint, in which we calculated the distance to each feature, applied a decay function, assigned weights to different feature classes and aggregated into a single footprint metric. This method is described in more detail in the supplementary methods, along with sensitivity analyses using alternative calculations of the footprint.

Although we were primarily interested in the responses of animals to the human footprint, we included additional spatial covariates in our habitat selection models to account for the effect of natural environmental features on space use, all at a 30 m resolution. We chose a suite of covariates *a priori* that we expected to be important for the species in our analysis, including elevation, slope and the distance to five land cover types (forest, herbaceous, scrub, barren and water). We obtained rasters of slope and elevation from the National Elevation Dataset, using the FedData R package [50]. We used the US Geological Survey National Land Cover Database (NLCD [51]) to determine land cover in each study area, combining NLCD classes into five broad land cover types (electronic supplementary material, methods) and generating distance rasters for each. Prior to modelling, we centred and scaled the values of elevation and slope for each population, such that the mean was 0 and standard deviation was 1. For the land cover distance covariates, we transformed distance with an exponential decay function (*y* = e^–*x*/750^) and then centred and scaled the values. This decay function was parameterized such that it drops steeply for 500 m and reaches 5% of its initial value at 2.25 km. We tested covariates for correlation, and of the 560 pairwise comparisons across all 20 populations, only four covariate pairs showed high correlation (|*r*| > 0.7). However, model estimates were stable with no issues of identifiability, so we retained all covariates for all populations to facilitate comparability.

### (c) Statistical analyses

#### (i) Resource selection functions

Using RSFs, we compared each animal’s GPS locations (‘used’ points) to available points within that animal’s home range. We calculated 95% kernel utilization distributions (KUDs) to approximate the home range of each individual animal in each period (2019 and 2020), using the 'amt' R package [52]. Here, we use the term ‘home range’ to represent the area used by the animal during the short study duration, recognizing that our home range estimates do not capture the true home range over a lifetime. We randomly sampled available points within the home range, with a 1 : 50 ratio of used : available points. We then extracted the value of each covariate at all points.

We fitted RSFs with generalized linear mixed models that included population-level hierarchical effects (e.g. [53,54]), with parameters estimated through a Bayesian framework. We ran a separate model for each population (park-species) with an identical modelling framework for all species. Model covariates included human footprint, elevation, slope and distances to five land cover types: forest, herbaceous, scrub, barren and water. We allowed the intercepts and all selection coefficients to vary across individual-years, and individual coefficients were drawn from population distributions. We also estimated posterior distributions for the population mean footprint selection coefficient for 2019 and for 2020 and for the interannual difference in population means.

Our binary response variable was ‘used’, equal to 1 if the location was used by the animal and 0 if available in the home range. We modelled this response as a Bernoulli random variable, where *p* is the probability of use of location *i*, such that *used*_*i* _~ Bern(*p_i_*). The linear predictor was modelled with a logit link function, where each *β* coefficient was allowed to vary by individual-year *j*:


$$\begin{array}{rl} \text{logit}(p_{i}) & =\beta_{\text{intercept}[j]}+\beta_{\text{footprint}[j]}*{\text{footprint}}_{i}+\beta_{\text{elevation}[j]}*{\text{elevation}}_{i}+\beta_{\text{slope}[j]}*{\text{slope}}_{i}\\ & +\beta_{\text{barren}[j]}*{\text{barren}}_{i}+\beta_{\text{forest}[j]}*{\text{forest}}_{i}+\beta_{\text{herbaceous}[j]}*{\text{herbaceous}}_{i}+\beta_{\text{scrub}[j]}*{\text{scrub}}_{i}\\ & +\beta_{\text{water}[j]}*{\text{water}}_{i}. \end{array}$$

We derived individual-level selection parameters from a population-level hyperdistribution, where the hyperparameters specified the mean response (*μ_β_*) and variation among individuals (precision: *τ_β_*; inverse of variance *σ*^2^). Individual coefficients were modelled as: *β_j_*Normal(*μ_β_, τ_β_*). For the five land cover coefficients, we jointly modelled the precision matrix using a multivariate normal distribution.

We assigned weakly informative prior distributions for population hyperparameters. For all population means (*μ_β_*), we used a normal distribution with a mean of 0 and precision of 0.01. For the intercept population precision parameter (*τ*_*β*intercept_), we derived the prior distribution by transforming a uniform prior for standard deviation (*σ*) from 0 to 100, where *τ* = 1/*σ*^2^. Population precision parameters (*τ_β_*) for the footprint, slope and elevation coefficients followed uniform priors from 0 to 25. For *τ_β_* population precision for the land cover coefficients, we used a Wishart distribution with 5 × 5 identity matrix to describe inverse scale, and 6 degrees of freedom, corresponding to uniform priors on correlations in selection among all pairs of land cover types. We only estimated effects for the five land cover distance covariates for individuals with that cover type within their home range by multiplying the selection coefficient by an indicator variable (1 = land cover type present, 0 = land cover type absent).

We estimated the posterior distributions of all parameters using Markov chain Monte Carlo sampling with JAGS, implemented in R with the package rjags [55]. We ran three chains, each with 30 000 iterations, a discarded burn-in of 3000 iterations and a thinning rate of 3. For all models, we examined traceplots and used the Gelman–Rubin Ȓ statistic to evaluate chain convergence, where values <1.1 indicate convergence. For all covariates, we inferred selection and avoidance with positive and negative beta coefficients, respectively, if the 95% credible intervals did not overlap zero.

#### (ii) Individual change in selection

We were interested in how individual animals changed their selection of the footprint from 2019 to 2020, and how this change varied across individuals as a function of their exposure to human disturbance. For the subset of individuals tracked in both years, we ran a model in which the response variable was the individual change in the footprint selection coefficient from 2019 to 2020, and the predictor was the mean footprint in the individual’s 2019 home range (exposure2019*_j_*). We estimated a separate intercept (*β*_intercept[_*_k_*_]_) for each population (*k*), each with a normal prior distribution with a mean of 0 and precision of 0.01. We assigned the slope parameter (*β*_slope_) a normal prior distribution with a mean of 0 and precision of 0.01. We defined the linear predictor for the change in footprint selection coefficient for each individual *j* as:


$$\Delta \beta_{\text{footprint}[j]}=\beta_{\text{intercept}[k]}+\beta_{\text{slope}}*\text{exposure}2019_{j}.$$

The likelihood was expressed as *μ*_Δ*β*footprint[*j*] _~ Normal(Δ*_β_*_footprint[_*_j_*_]_, *τ*_Δ*β*footprint[*j*]_), where *μ*_Δ*β*footprint[*j*]_ was the mean of the posterior distribution of the change in individual footprint selection coefficient, as estimated in the RSF. The precision (*τ*_Δ*β*footprint[*j*]_) was calculated from the standard deviation of this posterior distribution (*τ*_Δ*β*footprint[*j*]_ = 1/*σ*_Δ*β*footprint[*j*]_^2^).

#### (iii) Functional response models

For all collared animals, we also assessed how exposure to the human footprint-mediated individual selection or avoidance of the footprint by considering a functional response in resource selection (i.e. change in selection as a function of variation in availability [53,56]). We also compared how this functional response varied between 2019 (normal visitation) and 2020 (park closures). We ran Bayesian linear models where the response variable was the individual-year beta selection coefficient for footprint as estimated from the RSF, and the explanatory variable was the mean footprint value of all available locations in that individual-year’s home range (95% KUD). We estimated a separate slope for 2019 and 2020.

In our first functional response model (global model), the slopes for each year were the same across all parks and species, with a separate intercept for population. We also fitted a second model (guild model) in which the slopes varied not only by year but by guild (large carnivore and ungulate; we excluded red fox, the only small carnivore). To explore species-specific functional responses to the footprint and to park closure, we fitted a third functional response model (‘species model’), in which we estimated a separate slope and intercept for each species. In these models, we accounted for the uncertainty in estimated footprint selection coefficients by incorporating the posterior mean and standard deviation of each coefficient into the likelihood function, treating the coefficients as observed values with measurement error.

In the global model, we modelled the linear effect of mean human footprint in the home range of individual *j* (exposure*_j_*) on individual selection for the footprint (*β*_footprint[_*_j_*_]_). We estimated a separate intercept (*β*_intercept[_*_k_*_,_*_t_*_]_) for each population (*k*) and year (*t*), each with a normal prior distribution with a mean of 0 and precision of 0.01. We estimated a separate slope (*β*_slope_) for each year *t* and assigned this parameter a weakly informative normal prior distribution with a mean of 0 and precision of 0.01. We defined the linear predictor for the individual footprint selection coefficient as:


$$\beta_{\text{footprint}[j]}=\beta_{\text{intercept}[k,t]}+\beta_{\text{slope}[t]}*{\text{exposure}}_{j}.$$

The likelihood was expressed as *μ_β_*_footprint[_*_j_*_]_ ~ Normal(*β*_footprint[_*_j_*_]_, *τ_β_*_footprint[_*_j_*_]_), where *μ_β_*_footprint[_*_j_*_]_ was the mean of the posterior distribution of the individual footprint selection coefficient, as estimated in the RSF. The precision (*τ_β_*_footprint[_*_j_*_]_) was calculated from the standard deviation of this posterior distribution (*τ_β_*_footprint[_*_j_*_]_ = 1/*σ_β_*_footprint[_*_j_*_]_^2^). The guild and species models were similar, except that in the guild model we allowed the effect of footprint on selection to vary across guilds (separate slope for each guild in each year, with separate intercept for population-year), and in the species model we allowed the effect of the footprint on selection to vary across species (separate intercept and slope for each species in each year).

## 3. Results

Averaged across all 20 populations (229 individuals), animals overall avoided the human footprint in NPS protected areas to a similar degree during both the period of typical visitation in 2019 (mean *β*_footprint,2019_ = -1.88, 95% credible interval [CI]: −2.26 to −1.50) and the park closure in 2020 (mean *β*_footprint,2020_ = -1.75, 95% CI: −2.23 to −1.26). This pattern also held when averaging across all individual animals collared in each year (mean *β*_footprint,2019_ = -2.62, 95% CI: −2.94 to −2.29; mean *β*_footprint,2020_ = -2.15, 95% CI: −2.44 to −1.87).

**Figure 3.**
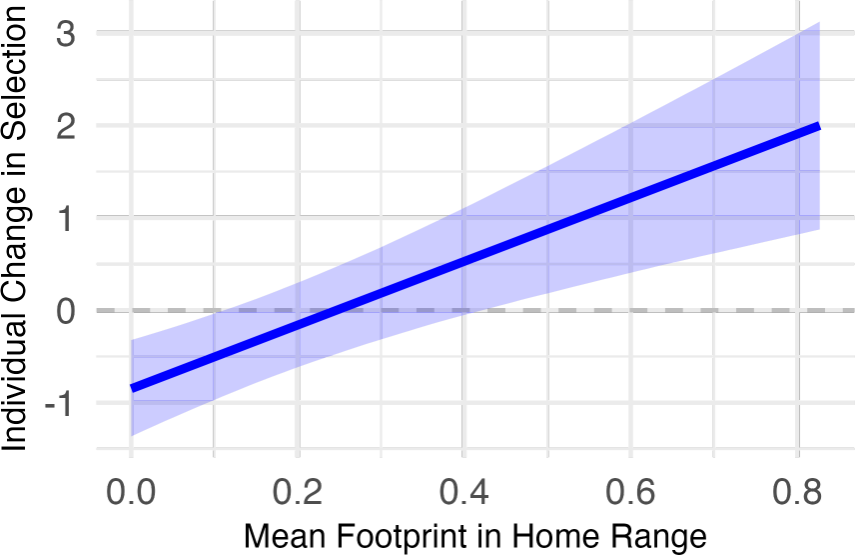
Change in footprint selection for all individual animals that were tracked in both 2019 and 2020, across all species and parks. Estimated relationship between the mean human footprint in an individual animal’s 2019 home range, and the difference in its footprint beta selection coefficient from 2019 to 2020. A positive value represents stronger selection (or weaker avoidance) of the human footprint when the park was closed to visitors, and a negative value represents weaker selection (or stronger avoidance). This model was based on resource selection function results for *n* = 117 individuals tracked in both years.

For individuals collared in both years, there was no consistent directional change in the beta selection coefficient from 2019 to 2020 (mean change across individuals = 0.483, 95% CI: −0.003 to 0.971). However, we found that the mean footprint in an individual’s home range (in 2019) predicted the extent to which it changed its selection for the footprint during park closure (figure 3). Individuals in more developed areas increased their selection (or decreased their avoidance) of the footprint during park closure, while those in more remote areas showed a smaller change in the opposite direction.

**Figure 4.**
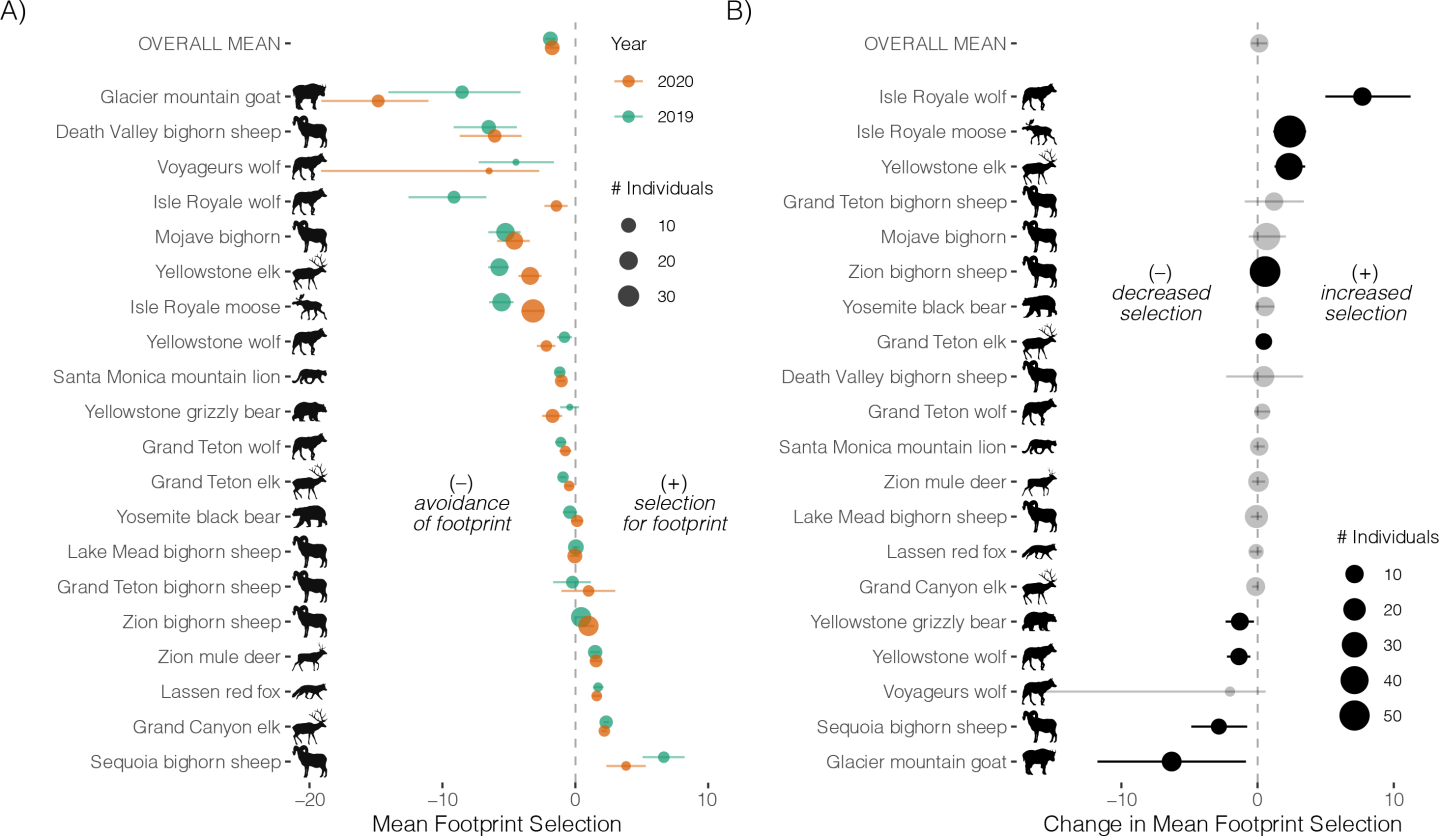
Population-level responses to the human footprint across US national parks (2019−2020) and responses to park closures during the 2020 pandemic. (A) Points represent the population mean selection coefficient for the human footprint in 2019 (green) and 2020 (orange). Negative values indicate that, on average, the individuals in the population avoided the human footprint, and positive values indicate that they selected for it. (B) Points represent the difference in the population mean selection coefficient for the human footprint from 2019 to 2020. Positive or negative values respectively indicate that, on average, the individuals in the population increased or decreased selection for the human footprint when parks were closed in 2020. Estimates in black have 95% CIs that do not overlap 0, indicating a meaningful difference in selection from 2019 to 2020. Error bars correspond to 95% credible intervals. The size of the points scales with the number of individuals in each period. Species icons from PhyloPic.org.

Populations varied in their mean selection of the human footprint and in responses to the park closure. Of the 20 populations, 11 on average avoided the human footprint in both years (95% CIs not overlapping 0), and four selected the human footprint in both years (figure 4A). For 11 of 20 populations, the 95% CI of the difference in population mean footprint selection from 2019 to 2020 overlapped 0, indicating a limited population-level response to the park closures. For the remaining nine populations, the mean selection coefficient varied between 2019 and 2020, with five populations increasing their selection of the footprint (or decreasing their avoidance of the footprint) in 2020 relative to 2019, and four showing the opposite pattern (figure 4B).

**Figure 5.**
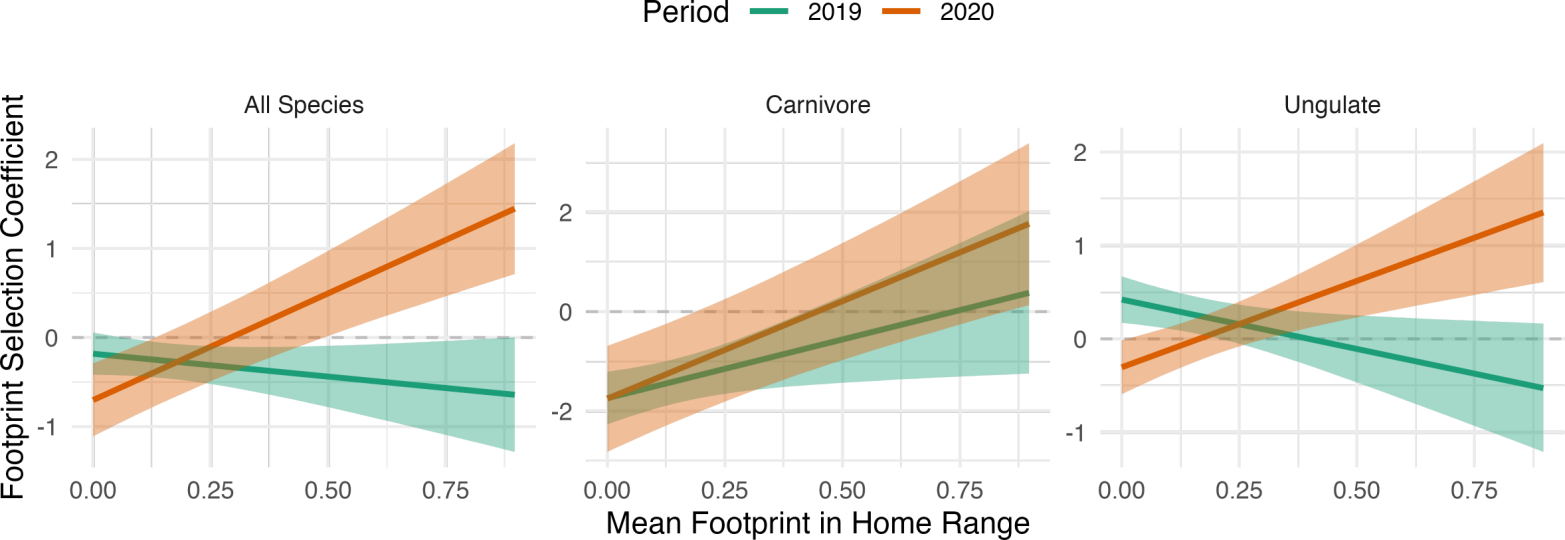
Selection of the human footprint by mammals in US national parks (2019−2020) varied with exposure and park closure. Graphs represent the predicted relationship between the mean human footprint in an individual animal’s home range and its avoidance/selection of the human footprint (as represented by individual beta coefficients in the resource selection function). This functional response varied between 2019, when the parks experienced typical patterns of visitation, and 2020, when the parks were closed to people. In these models, a single slope was estimated for each year, and the intercepts varied by population and year (average values are plotted below). The left panel displays the predictions of the global model (all species combined), and the centre and right panels display the predictions for the guild model (separate functional responses for large carnivores and ungulates).

**Figure 6.**
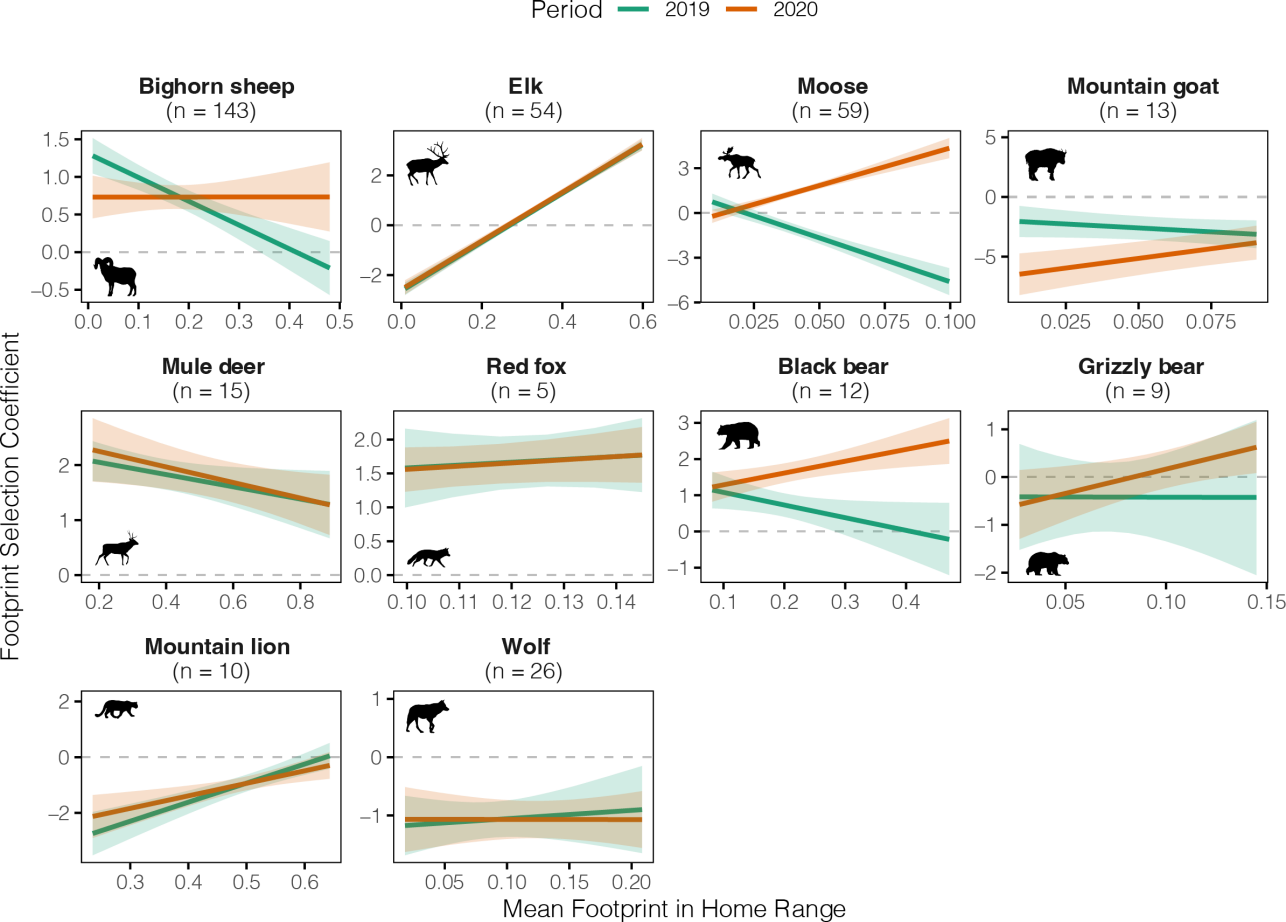
Species-specific functional responses to human footprint in US national parks (2019−2020). Predicted relationship between the mean human footprint in an individual animal’s home range and its avoidance/selection of the human footprint, estimated separately for each species and year. Sample sizes indicate the number of individual-years per species. A slope of zero indicates that animals exhibit a consistent response to the human footprint, regardless of how much of it is in their home range. A negative slope indicates sensitivity to human disturbance, as animals with more footprint in their home range increasingly avoid footprint (or select for it less). A positive slope indicates habituation to human disturbance, as animals with more footprint in their home range avoid footprint less (or select for it more). In 2020, a positive slope may also indicate that animals selected more for the human footprint given the absence of humans in these areas. Note that both the *x*- and *y*-axis scales change across plots; the *x*-axis scales reflect the range of mean footprint values observed for each species. Species icons from PhyloPic.org.

When aggregating across all individuals, variation in footprint selection could be explained by a functional response, in which individual selection of the footprint varied as a function of the mean footprint within the individual’s home range (figure 5). Overall, in 2019, there was a weakly negative effect of footprint exposure on footprint selection, which switched to a positive effect in 2020, such that individuals with a higher mean footprint in their home range selected positively for the footprint during park closures. The pattern for ungulates was similar to the overall pattern (and likely driving this overall pattern), while large carnivores showed a weakly positive functional response in 2019 (although predicted mean selection was negative across all values of the footprint) and a steeper functional response in 2020 (figure 5). However, there was variation in this functional response across species, and for many species, the slope of the functional response did not change between 2019 and 2020 (figure 6).

## 4. Discussion

Our synthesis of GPS tracking data of mammals across 14 US NPS protected areas revealed diverse and nuanced effects of both human presence and recreation infrastructure on resource selection by animals. When averaging across all individuals in our study and accounting for features of the natural environment that shape habitat selection, animals generally avoided the human footprint during time periods of typical visitation, indicative of a perception of risk in these areas. During the COVID-19 pandemic of 2020, visitation of NPS protected areas abruptly ceased, eliminating a major disturbance and source of perceived risk for mammals, but there was no detectable effect of park closure on the overall magnitude of mean avoidance of the human footprint by mammals in our study.

At first glance, this overall similarity in avoidance of the human footprint regardless of whether parks were open to visitors might suggest a lack of plasticity in behavioural responses to human disturbance or indicate that avoidance of the footprint is more related to low habitat suitability in these areas than to human presence *per se*. However, responses both to the human footprint and to the park closure varied across populations, species and individual animals. Some of this variation was explained by baseline levels of exposure to human disturbance: animals exposed to a higher human footprint in their home ranges showed the greatest change in selection for the footprint from 2019 to 2020. These animals in more developed areas switched from avoiding the human footprint when the parks were open to visitors to a neutral or positive association with the human footprint in the absence of visitors.

### (a) Variable responses to human footprint and presence

Some populations avoided the human footprint consistently, regardless of whether people were present or not, including the Mojave NPRES and Death Valley NP bighorn sheep, the Santa Monica NRA mountain lions and the Yellowstone NP wolves, consistent with other studies [29,57,58]. Other species exhibited more flexibility in response to park closures, perhaps to exploit habitat near developed areas when humans were absent. For example, the Yosemite NP black bears switched from avoidance to selection of the human footprint when the park was closed in 2020. Collared black bears in our study largely lived in Yosemite Valley, a highly developed area popular with visitors, where bears are frequently aversively conditioned by managers, and bears apparently took advantage of the absence of people to access the abundance of natural foods in the valley. The Isle Royale NP moose and wolves also responded to the closures; they generally avoided the human footprint in both years, but less so when the park was closed. These results echo findings from a camera trap study that detected more moose on Isle Royale trails in 2020 as compared with 2021 [59].

Although many wild animals are wary of humans, some reap some benefit from human activities: human disturbance can offer protection to prey and smaller carnivores from predators that avoid humans (i.e. a human shield [25,60]). This phenomenon may explain why Zion NP mule deer selected the human footprint in both years. Notably, this sample of collared deer occupied areas with a high human footprint and likely were habituated to human presence [61]. In Glacier NP, mountain goats use areas of higher human activity for protection from their predators and for mineral subsidies found in human urine [62]. These mountain goats avoided the human footprint in both years, but more strongly in 2020, perhaps because there was no longer any benefit associated with the footprint in the absence of human presence. Perceptions of anthropogenic risk may be less plastic than perceptions of reward, as predicted by theory that animals should prioritize risk avoidance given the high fitness costs of predation [63].

Some species showed a positive functional response to the human footprint, indicative of habituation and consistent with other studies of animals in NPs [29]. Collectively, in both 2019 and 2020, elk in more remote areas avoided human footprint, whereas elk in more developed areas selected it. For some species, the nature of the functional response varied depending on whether the park was open or closed. For the Isle Royale NP moose and Yosemite NP black bear, there was a negative functional response when parks were open: animals in areas of higher footprint avoided the footprint more within their home range. In 2020, however, the functional response slope changed, whereby animals in areas of higher footprint selected the footprint, matching the overall trend observed when averaging across species.

The differential effects of human activity on mammal species have the potential to alter interspecific niche partitioning [64,65], potentially altering predation and competition and reshaping ecological dynamics [66,67]. However, there is limited and mixed evidence for the impacts of recreation on species spatiotemporal overlap [59,68] and the cascading consequences of human-induced behaviour change [14,69]. Future multispecies research may shed light on the pathways through which recreation alters species interactions [62].

### (b) Alternative scales of human avoidance behaviour

Our analysis quantified the effects of the human footprint on animals' habitat selection within their home ranges (i.e. third-order selection), but it is possible that animals respond to the human footprint and presence through other behavioural mechanisms. For example, although we found no effects of closure on the Santa Monica NRA mountain lions, another study of their movement found that they used smaller areas and moved shorter distances during the pandemic shutdown as compared with just before [70]. Animals may also avoid human activity in time, rather than space, to take advantage of habitat in proximity to human disturbance while minimizing temporal overlap [71]. The coarse resolution of many of our datasets precluded analysis of temporal variation in resource selection or of fine-scale movement decisions through methods like step selection functions [72].

Notably, our analysis was restricted to individuals and populations that overlapped spatially with the human footprint in protected areas. Many animals had home ranges far from disturbance, and these were dropped from the analysis, including the entire dataset on Yukon–Charley Rivers NPRES wolves. Some animals avoid human disturbance via second-order selection (i.e. selection of a home range from the larger landscape [73,74]), preferentially establishing home ranges in more remote areas, or may not have any direct exposure to regular human activity. Even if human disturbance plays no role in second-order selection, the location of many animals’ home ranges in the expansive undeveloped backcountry of NPS protected areas frees them from the need to respond to the human footprint within their home ranges. Indeed, theory predicts that animals should respond to the most important factors limiting their fitness at coarser scales (e.g. second-order selection) in accordance with the limiting factors avoidance hypothesis [75].

Our study adds to a growing literature of the impacts of the 2020 pandemic ‘Anthropause’ on wildlife populations. Although some individuals and populations responded strongly to the absence of people during park shutdowns, most did not. With the relatively short duration of park closures, many animals may not have had enough time to perceive and respond to the change in human activity, particularly those with low exposure to human development in their home ranges. Given the strong fitness benefits of risk avoidance behaviour, animals often retain such behaviours even when the risk is gone [76].

### (c) Balancing recreation and conservation

Although the spatial extent of the built environment in most NPS protected areas is minimal, avoidance of the human footprint by mammals may exclude animals from otherwise usable habitat and reduce the conservation capacity of protected areas. However, the low and concentrated human footprint in much of NPS-managed land likely minimizes the overall impact on wildlife in parks. Notably, many of the animals in the study did leave the boundaries of protected areas at some point, highlighting the need to consider the impact of human land uses outside parks on the species within them, particularly for wide-ranging and migratory species [77]. Furthermore, our findings suggest that some animals habituate to benign recreation, thereby limiting its detrimental effects, although potentially setting the stage for human–wildlife conflict (e.g. vehicle collisions, property damage) in areas of high human and wildlife activity.

In addition to the trade-offs associated with human disturbance, animals balance many other spatially variable risks and opportunities in protected areas. The outsized role of these other factors may explain the relatively small effect of disturbance on habitat selection of some animals, particularly in areas where disturbance is limited in magnitude and extent. For example, a recent study of 17 years of grizzly bear movement in Yellowstone NP found that the restriction of recreation in Bear Management Areas had limited effects on bears, with resource availability apparently playing a larger role in bear space use [78]. In these cases, the persistence of sensitive species in protected areas may depend more on access to resources, necessitating prioritizing habitat management over visitor management.

Although the 2020 COVID pandemic and associated forced shutdown of NPs during the 2020 COVID had generally devastating consequences [79], it presented a useful pseudonatural experiment to better understand wildlife responses to human activity as differentiated from human footprint. However, the experimental treatment of removing people from the landscape may not be an appropriate way to understand the conservation implications of expanding human development into natural habitats. In the ‘Anthropause’, risk-averse individuals and species may have already been displaced prior to the experimental treatment of human removal, while those individuals with a high exposure to humans had previously been habituated. These changes due to the initial encroachment of people may be large [80] relative to subsequent modulations in the number of people in an already developed landscape.

## 5. Conclusion

Both inside and outside protected areas, animals must navigate perceived risks and rewards associated with human disturbance. Our study contributes to a growing body of evidence that these risk–reward trade-offs vary across species, populations and individuals, leading to heterogeneity in spatial responses of animals to the human footprint. While some animals can habituate to benign human presence or exhibit behavioural flexibility depending on the level of human activity, others are more sensitive to human disturbance regardless of the presence of humans or history of exposure. Further understanding of this heterogeneity can enhance understanding of the role of human disturbance in filtering wildlife communities and inform multi-species conservation alongside recreation in our protected areas.

## Data Availability

The datasets analysed in this study are available in the Dryad Digital Repository [81]. While we do not share raw location data owing to the sensitivity of animal locations, we provide complete information on the format of data files, intermediate data products, and the scripts necessary to reproduce analyses. Supplementary material is available online [82].
